# Ambient ultraviolet radiation induces DNA damage in amphibian larvae under semi-natural conditions

**DOI:** 10.1242/jeb.251017

**Published:** 2025-11-19

**Authors:** Coen Hird, Rebecca L. Cramp, Craig E. Franklin

**Affiliations:** School of the Environment, The University of Queensland, Brisbane 4072, Australia

**Keywords:** Amphibian declines, Ecophysiology, Temperature, Mesocosm

## Abstract

Ambient sunlight contains ultraviolet radiation (UVR), a potent genotoxic stressor, yet whether natural UVR induces DNA damage in free-living amphibian larvae remains unclear. We reared striped marsh frogs (*Limnodynastes peronii*) in outdoor mesocosms under low versus high ambient UVR treatments for 5 days and quantified cyclobutane pyrimidine dimers (CPDs) by ELISA. Measurable CPD levels were detected under both UVR exposures, providing direct evidence that natural sunlight induces DNA damage in amphibian larvae. Although mean CPD levels did not differ between treatments, quantile regression revealed higher levels of CPDs in both the lower (τ=0.05) and upper (τ=0.95) quantiles under high UVR. This pattern suggests that while average DNA damage levels were similar, subsets of individuals experienced disproportionately high CPD burdens under increased UVR. These results highlight the value of mesocosms as a tool to bridge laboratory and field contexts to evaluate the effects of UVR on aquatic fauna, particularly as UV environments shift with climate change.

## Teaser

**Summary:** Natural sunlight induces measurable DNA damage in amphibian larvae, with subsets of individuals disproportionately affected, underscoring the value of mesocosms for studying ecologically relevant impacts of increasing UV radiation under climate change.

## INTRODUCTION

Amphibians have experienced catastrophic global declines, making them among the most endangered vertebrates (Alford and Richards, 1999; Green et al., 2020; McCallum, 2007; Mendelson et al., 2006; Stuart et al., 2004). Climate change is expected to exacerbate these declines by altering multiple environmental stressors, including ultraviolet radiation (UVR), which remains elevated in parts of the world despite the success of the Montreal Protocol (Dhomse et al., 2019; Lemus-Deschamps and Makin, 2012; Madronich et al., 1995). UVR is predicted to change markedly in freshwater ecosystems under future climates (Bais et al., 2015, 2018; Barnes et al., 2019; McKenzie et al., 2011; Williamson et al., 2014).

UVR encompasses wavelengths from 100 to 400 nm and is typically divided into UV-C (100–280 nm, largely absorbed by the atmosphere), UV-B (280–315 nm) and UV-A (315–400 nm). While UV-B has received the most attention in amphibian research owing to its potent genotoxicity (Alton and Franklin, 2017), UV-A is up to 20-times more intense than UV-B in natural sunlight reaching terrestrial and aquatic environments (Londero et al., 2019). UV-B is highly effective at inducing direct damage to DNA, forming cyclobutane pyrimidine dimers (CPDs) and (6–4) photoproducts that disrupt gene expression, arrest cell cycles and trigger mutations or apoptosis (Cadet and Wagner, 2013; Goodsell, 2001; Tevini, 1993).

UVR is a potent genotoxic stressor, yet its direct effects on UV-sensitive organisms under natural exposure regimes remain poorly understood. Amphibians are among the most UV-sensitive vertebrates (Downie et al., 2023), with early life stages occurring in freshwater habitats that can often receive intense solar radiation. UVR exposure can elevate mortality and malformations, delay development, reduce growth, trigger physiological and behavioural changes, and impair locomotion in amphibian embryos and larvae (see review by Alton and Franklin, 2017). From laboratory studies, it is hypothesised that the detrimental effects of UVR on amphibians is in part driven by the downstream impacts of UVR-induced genotoxicity (Londero et al., 2019). However, whether ambient levels of solar UVR in the environment are sufficient to induce measurable DNA damage in free-living amphibians remains unclear.

Field experiments often yield conflicting results because of substantial variation in environmental conditions, including UVR transmittance, larval pigmentation and egg colouration (Licht and Grant, 1997). Mesocosms provide a bridge between lab and field by capturing naturalistic variation while maintaining a level of experimental control (Boone and James, 2005; Fordham, 2015). Most mesocosm studies of UVR effects on amphibians have reported reduced survival or growth in amphibians exposed to naturalistic or elevated UVR, either in isolation (Belden and Blaustein, 2002; Ovaska et al., 1997; Searle et al., 2010) or in combination with other stressors (Britson and Threlkeld, 2000; Hatch and Blaustein, 2003; Levis and Johnson, 2015). However, many of these studies either did not report the UVR irradiances at the substrate or reported very low levels (<10 µW cm^–2^), limiting mechanistic interpretation. Without physiological markers such as DNA damage, it is difficult to determine whether reported effects stem from direct UVR exposure or from indirect environmental changes (e.g. altered temperature, oxygen levels or resource availability).

Despite widespread recognition that UVR is an important natural stressor, no mesocosm studies have directly tested whether natural sunlight exposure regimes induce DNA damage in amphibian larvae. In this study, we manipulated the incident UV levels in semi-natural mesocosms that allowed striped marsh frog (*Limnodynastes peronii*) larvae to behaviourally avoid natural UVR. We hypothesised that larvae raised in mesocosms would exhibit measurable CPDs, consistent with laboratory UVR exposure and that *L. peronii* exposed to natural, Austral summer levels of UVR would have higher levels of DNA damage compared with larvae from mesocosms with some shielding from ambient UVR.

## MATERIALS AND METHODS

### Study species

*Limnodynastes peronii* (Duméril and Bibron 1841) were chosen as a known UVR-sensitive species (Alton and Franklin, 2017; Kern et al., 2014; Morison et al., 2020; van Uitregt et al., 2007). This frog is known to breed in ephemeral ponds (Kern et al., 2014) and is listed as least concern on the IUCN Red List (IUCN SSC Amphibian Specialist Group, 2022). Spawn was collected under the Queensland Department of Environment and Heritage Protection Scientific Purposes Permit (WA0017092) and procedures were approved by The University of Queensland's Animal Ethics Unit (2021/AE000132).

### Mesocosms

**Fig. 1.**
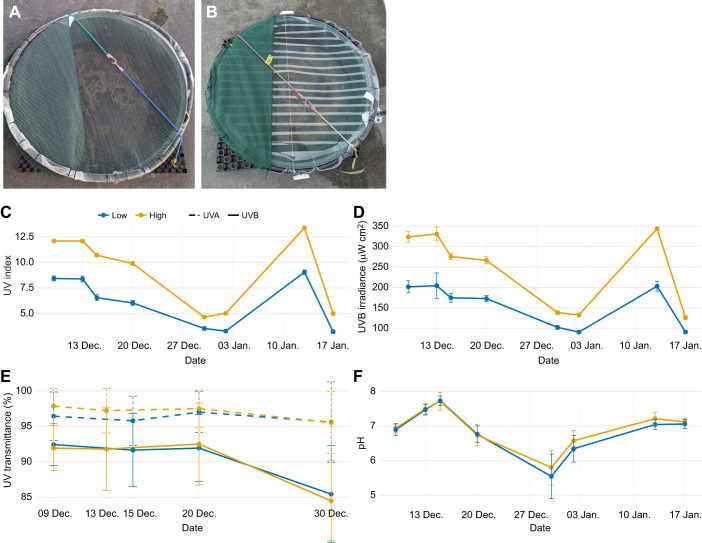
**UVR treatments and *in situ* conditions in outdoor mesocosms.** (A,B) Overhead photographs of mesocosms showing treatment roofs: high UVR (100% ambient UVR) with no UV-B film (A) and low UVR (70% ambient UVR) with separated strips of UV-B-blocking film fixed to the roof (B). Polyester has a sharp UV cut-off (near-zero transmission <∼314 nm, partial 320–340 nm, high transmission through most UV-A and visible). (C,D) Time series of UVI (C) and UV-B irradiance (µW cm^–^²) (D) measured at the water surface within 2 h of solar noon using handheld radiometers. At each date, values are treatment means from *n*=4 mesocosms per treatment, each the mean of 10 readings per mesocosm; error bars show ±s.d. The low UVR treatment reduced mean UVI by ∼30% and mean UV-B by ∼36% relative to high UVR. (E) Percentage transmittance of mesocosm water measured with a UV-VIS spectrophotometer at 300 nm (UV-B, solid) and 360 nm (UV-A, dashed); points are treatment means±s.d. (all mesocosms). (F) pH of mesocosm water over time (all mesocosms); points are treatment means±s.d. Colours are consistent across panels: high (orange) and low (blue).

Experimental mesocosms (PTT12W, Rapid Plas, Tamworth, NSW, Australia; 250 litres, 1000 mm diameter×420 mm height, green UV-stabilised polyethylene) were placed on the roof of a building at The University of Queensland (St Lucia, Magandjin, Australia). Mesocosms were covered with a thin white mesh to exclude animals, leaf litter, and other extraneous material. All mesocosms were also one-third covered with 70% light blocking shade cloth (green) to give larvae access to shade. Two mesocosms were set aside to hatch spawn and were entirely covered with 70% light blocking shade cloth (white). All mesocosms were filled with 150 litres rainwater up to an overflow hole and then inoculated with 1 litre of pond water in July 2021, collected from a known breeding site for *L. peronii* nearby. The purpose of this was to introduce natural microorganisms into the mesocosm environment to increase ecological realism and provide larval food. All mesocosms contained ∼1 cm of cleaned gravel at the base, along with two larger rocks and two *Myriophyllum propinquum* (common milfoil) plants. UVR treatments (Fig. 1A,B) were created by applying Melinex polyester UV-B blocking film (Melinex^®^ 516 polyester, 100 µm, Archival Survival, Doncaster, VIC, Australia) to the roof of the 70% UVR mesocosms. Polyester has a well-defined UV cut-off: manufacturer data show nearly zero transmission below ∼314 nm, partial transmission in the ∼320–340 nm range and high transmission (∼80–90%) through most UV-A and the visible spectrum. It was assumed that incident UVR scattered upon entering the water, creating a diffuse UVR exposure at the substrate, where larvae were most often observed. We did not quantify the full UV exposure spectra and acknowledge that the UV-B blocking film may also mildly attenuate UV-A; accordingly, we report UVI and UV-B to benchmark treatment differences and we interpret genotoxicity primarily in relation to UV-B-driven CPDs.

UVI is a standardised, wavelength-weighted metric that integrates both UV-A and UV-B irradiance according to their potential to cause erythema (sunburn) in human skin (World Health Organization et al., 2002). Originally designed for public health, this widely accessible tool can be used to assess UVR's broader biological impacts and, being calibrated under natural sunlight, is well-suited for measuring and comparing outdoor UVR levels (World Health Organization et al., 2002). In this study, we used both UVI and unweighted UV-B measurements to characterise UVR treatments. Because UVI weighting favours UV-B effects, it may under-represent the contribution of UV-A to oxidative stress in non-human organisms. However, UV-B was found to be more detrimental to vertebrates than UV-A in a global meta-analysis (Downie et al., 2023).

Part clutches of freshly laid *L. peronii* (*n*=5) spawn were collected on Yuggera Country (Brisbane, Queensland, Australia) on 1 December 2021 and transferred immediately to a mesocosm covered completely with 70% UV-blocking white shade cloth and filled with 150 litres rainwater inoculated with 500 ml of pond water from the collection site. Clutch ID was not tracked throughout the study because of logistical constraints, so it was assumed that any interclutch variation was randomly distributed across treatments. Temperatures were logged using iButtons (type DS1921H; Maxim/Dallas Semiconductor Corp., USA). Upon hatching on 4 December, spawn were left to develop to Gosner stage 25, which all reached by 8 December. On 10 December, larvae (6 days post-hatch) were randomly transferred to established mesocosms (*n*=50 larvae per mesocosm in 12 mesocosms; *n*=63 larvae per mesocosm for 6 mesocosms for future CPD sampling, split evenly across treatments). Mesocosms were covered or not covered with UV-B blocking film to create either ∼70% (low) or ∼100% (high) UVR treatments, respectively. Low UVR treatments were achieved by fixing thinly separated strips of UV-B blocking film to the roof of the mesocosm (Fig. 1A,B).

To verify UVR treatment levels, handheld UVI (Solarmeter 6.5, Solarlight Company Inc., Glenside, USA) were measured at the water surface within 2 h of solar noon, every few days (Fig. 1C,D). On a clear benchmarking day (15 November 2021, 14:45 h), the high UVR treatment had a UVI of 4.07±0.12 (mean±s.d.; range 3.8–4.3), whereas the low UVR treatment measured 2.69±0.84 (range 1.5–3.8), i.e. 66% of the high treatment. Across the experiment, the low UVR treatment averaged ∼30% lower UVI and ∼36% lower UV-B than high UVR conditions (Fig. 1C,D).

During the experimental period, water temperatures were very similar across treatments. Mean daily temperature (±s.d., calculated across mesocosms) was 27.12±0.29°C in the high UVR treatment and 26.59±0.31°C in the low UVR treatment. Daily minima averaged ∼22–23°C and maxima reached ∼35–37°C across both treatments. These values confirm that water temperatures were highly consistent between treatments and remained within a similar thermal range throughout the experiment.

Larvae were sustained off the algae present in the mesocosms, and no further food was provided. Mesocosms were monitored every few days which included measuring water depth and collecting water samples to measure percentage UVR transmittance and pH (Fig. 1E,F), and measuring UVI and UV-B (IL1400BL, International Light Inc., Newburyport, USA) within 2 h of midday when the sky was least cloudy. UVR readings were averaged from ten readings taken at water level. Across the experiment, UVI averaged 9.80±3.03 in high-UVR and 6.48±2.10 in low UVR (34% lower in low UVR; ranges 4.4–13.5 and 2.9–9.3, respectively). Water pH was similar between treatments (high: 7.38±0.21, range 6.1–8.4; low: 7.38±0.14, range 6.4–8.5). Water was highly transmitting at both wavelengths measured: 300 nm (UV-B) transmittance was 91.08±4.46% (79.4–100%) in the high UVR treatment and 91.32±4.27% (75.9–100%) in the low UVR treatment, and 360 nm (UV-A) transmittance was 97.29±2.06% (89.5–100%) versus 96.35±3.06% (87.2–100%).

On 15 December (11 days post-hatch including 5 days of UVR exposure), the mesocosms containing 63 larvae (*n*=3 mesocosms per treatment) were sampled. A subset of these larvae (*n*=13 per mesocosm; *n*=78 total) were collected using nets. These larvae were collected at 13:00 h, during peak UVR. All larvae were Gosner stages 26–30 (Gosner, 1960) and no difference in developmental rate was detected between different mesocosms at this point. Larvae were euthanised in MS222 on ice immediately and blotted dry under red light to minimise DNA photorepair. Wet body mass was recorded and larvae were stored at −80°C until assaying. Separately, on 30 December, larvae remaining in mesocosms (originally stocked at 50–63 larvae per mesocosm) were counted and weighed to provide treatment-level estimates of survival and growth 26 days post-hatching.

Genomic DNA (gDNA) was extracted and purified from *L. peronii* whole-body homogenates using a Qiagen DNeasy Blood and Tissue Kit (Qiagen Inc., Hilden, Germany) and quantified with a Qubit dsDNA High-Sensitivity Assay Kit (ThermoFisher Scientific Inc., Waltham, MA, USA). CPD concentrations were quantified using an anti-CPD ELISA following the primary antibody manufacturer's protocol (Mori et al., 1991; NM-ND-D001, clone TDM-2, Cosmo Bio Co., Ltd.) modified to a protocol established in our previous studies (Hird et al., 2022, 2023a, 2024b), with 2% fetal bovine serum in phosphate buffered saline used as the blocking buffer. CPD concentration is reported as units of UV-C dose equivalents per 20 ng DNA.

### Statistical analyses

All analyses were done in the R statistical environment (v.4.3.0; r-project.org). Regression diagnostics were analysed using the performance package (v. 0.10.3; Lüdecke et al., 2021). Models were two-tailed and assumed a Gaussian error structure. Alpha was set at 0.05 for all tests.

Wet body mass was compared between mesocosm treatments using two-sample *t*-tests. DNA damage was modelled using a linear mixed model from the *lme4* package (v.3.1-3; https://CRAN.R-project.org/package=lme4; Bates et al., 2015) using UVR-blocking treatments as the explanatory variable, wet body mass as a covariate and mesocosm ID as a random effect. Contrasts were made using Type II Wald chi-square tests. Beyond comparing means, we examined whether UVR shifted the distribution of CPDs, including potential changes in both upper and lower tails. Elevated DNA damage, which results from exceeded DNA repair capacity, would increase upper quantile CPDs, while behavioural refuge use could reduce lower quantile CPDs. To test these possibilities, we fit quantile regressions (Hird et al., 2024a) on the 5th, 50th (median) and 95th percentiles (τ=0.05 and 0.95) using the *quantreg* package (v.5.95; https://CRAN.R-project.org/package=quantreg). Standard errors were obtained by nonparametric bootstrapping (50,000 resamples). *P*-values for τ=0.05 and τ=0.95 were adjusted for multiple testing using the Holm procedure, while the median regression was included for descriptive comparison. Quantile regressions were fitted at the individual level. We considered mixed-effects quantile regression, but with only six mesocosms (three per treatment) the random-effects variance was poorly identified. Therefore, we opted for fixed-effects models with robust bootstrap inference.

## RESULTS AND DISCUSSION

Survival of *L. peronii* to 26 days post-hatching was low in both treatments (mean±s.d.: 1.4±2.35 in the high UVR treatment and 2.75±4.97 in the low UVR treatment). There were no differences in wet body mass between UVR mesocosm treatments in *L. peronii* (*t*_48_=0.83, *P*=0.41). Larvae developed cyclobutane pyrimidine dimers (CPDs), confirming that UVR exposure in semi-natural conditions induces measurable DNA damage, despite opportunities for behavioural avoidance. Mean CPD concentrations (±s.d.) were 0.74±0.50 UV-C dose equivalents in the high UVR treatment and 0.55±0.32 in the low UVR treatment. Although CPD levels were lower than those observed previously under laboratory conditions (Hird et al., 2022, 2023a; Morison et al., 2020), they indicate that natural sunlight can induce DNA damage in free-living larvae. Given that CPDs can compromise genome integrity if unrepaired, these lesions may serve as a biomarker of UVR exposure in amphibians, although future research is needed to establish their ecological relevance. Because UVR sensitivity and repair dynamics can vary across early life stages (Crump et al., 1999; Smith et al., 2000), we sampled within a narrow window (Gosner 26–30), minimising ontogenetic effects on CPD accumulation. *L. peronii* larvae exposed to either low or high UVR for 5 days showed no significant difference in mean DNA damage (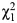
=2.26, *P*=0.13).

Many mesocosm studies contrast fully UV-B-shielded versus unshielded conditions (e.g. Searle et al., 2010; Hatch and Blaustein, 2003). While valuable, such ‘on/off’ designs may not capture the subtler shifts in incident UVR expected with interannual ozone variability, cloud cover or water clarity changes (see Bais et al., 2015; Barnes et al., 2019). Our manipulation (low versus high UVR) was chosen to approximate those realistic, percentage-level changes in exposure, while maintaining high absolute irradiance typical of clear, shallow habitats (UVI up to >13; Hird et al., 2025). By avoiding a fully shaded treatment, we aimed to test whether modest differences in ambient UVR translated into detectable differences in DNA damage.

The absence of treatment-level differences may reflect behavioural UVR avoidance, early-life acclimation reducing CPD accumulation, inter-individual variation in response, or a combination of the three. In previous laboratory work, we showed that low level UVR exposure did not induce a protective ‘UVR hardening’ response in *L. peronii* larvae (Hird et al., 2023a). However, the more complex and variable UVR cues in mesocosms may have enabled differing physiological or behavioural adjustments between individuals.

**Fig. 2.**
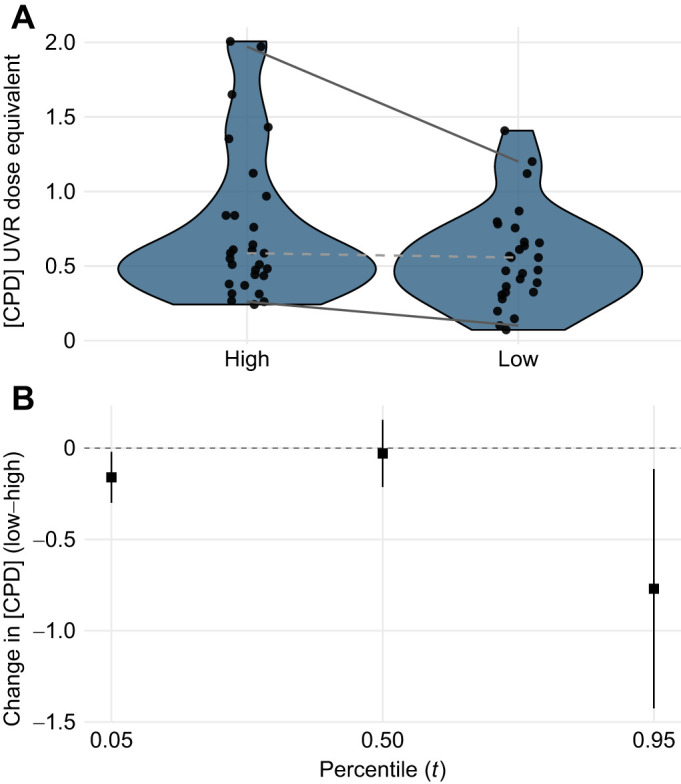
**DNA damage levels in the whole body of larval *Limnodynastes peronii* at midday after 5 days of UVR exposure in mesocosms with high versus low incident UVR.** (A) Cyclobutane pyrimidine dimer (CPD) concentration (data points) and violin plots for each mesocosm treatment. Lines are quantile regressions fitted at τ=0.05 (5th percentile), τ=0.50 (median) and τ=0.95 (95th percentile). The dashed line indicates the median regression (not significantly different from 0), solid lines indicate the lower and upper quantiles (significantly different from 0). High UVR: 100% ambient (*n*=28); low UVR: 70% ambient (*n*=29). (B) Estimated treatment effects (low–high UVR) from quantile regression at τ=0.05, 0.50 and 0.95, with bootstrapped 95% confidence intervals (50,000 resamples). Sample sizes are the same for both panels (high UVR *n*=28; low UVR *n*=29). Mean CPD concentrations (±s.d.) were 0.74±0.50 UV-C dose equivalents in the high UVR treatment and 0.55±0.32 in the low UVR treatment. *L. peronii* larvae sampled after exposure to either low or high UVR for only 5 days showed no significant difference in mean DNA damage (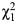
=2.26, *P*=0.13). At the 5th percentile, CPDs were slightly lower in the low UVR treatment (Δ=−0.16, 95% CI −0.30 to −0.02, Holm-adjusted *P*=0.047), whereas at the 95th percentile larvae from high UVR mesocosms showed substantially higher CPDs (∼30% higher; Δ=−0.77, 95% CI −1.42 to −0.12, Holm-adjusted *P*=0.047). The experiment was not replicated.

Indeed, targeted quantile regression revealed treatment differences at both distribution tails (Fig. 2). At the 5th percentile, CPD levels were slightly lower in the low UVR treatment (Δ=−0.16, 95% CI −0.30 to −0.02, Holm-adjusted *P*=0.047), whereas at the 95th percentile larvae from high UVR mesocosms showed substantially higher CPD levels (∼30% higher; Δ=−0.77, 95% CI −1.42 to −0.12, Holm-adjusted *P*=0.047). These results suggest that although average DNA damage levels did not differ significantly between treatments, a subset of individuals under full UVR accrued disproportionately greater CPD burdens. This pattern is consistent with inter-individual variation arising from behavioural avoidance, plasticity of physiological responses and/or clutch-to-clutch (genetic) differences. Maximum CPD levels may provide a sensitive biomarker of harmful UVR exposure in nature, especially if increases align with rising atmospheric UVR levels. However, whether elevated CPDs in a subset of larvae would influence population-level fitness or demography remains unclear. Longitudinal and interdisciplinary studies that track individuals through to metamorphosis could help clarify how such variation translates to ecological outcomes. Caution is also warranted when linking larval fitness solely to UVR-induced DNA damage. The uniformly low survival we observed across treatments underscores the difficulty of disentangling UVR effects from other environmental stressors in mesocosms. In this case, high peak water temperatures (>35°C) are a plausible driver of mortality, which may have masked smaller treatment-level differences in survival. However, few studies have explicitly tested for the combined effects of temperature and UVR. Higher water temperatures can alter DNA repair rates (Hird et al., 2022, 2023a; Langenbacher et al., 1997; Morison et al., 2020) and even moderate warming has been shown to buffer UV-B stress in high-altitude frogs (Tang et al., 2022). UVR exposure also influenced algal growth in some mesocosms, potentially affecting food availability, oxygen levels and opportunities for UVR avoidance. These interacting variables probably introduced considerable experimental noise. Yet this highlights the value of semi-naturalistic studies, which bridge the limited control of field experiments and the artificial simplicity of laboratory settings (Boone and James, 2005; Fordham, 2015). To better understand UVR effects in nature, future mesocosm work should systematically track co-varying factors such as temperature, algae and oxygen levels to assess their interactions with UVR exposure.
